# Application of ultrasonography in neonatal lung disease: An updated review

**DOI:** 10.3389/fped.2022.1020437

**Published:** 2022-10-25

**Authors:** Jin Wang, Hongjuan Wei, Hui Chen, Ke Wan, Ruifeng Mao, Peng Xiao, Xin Chang

**Affiliations:** ^1^ Department of Ultrasound Medicine, Nanjing Lishui People’s Hospital, Zhongda Hospital Lishui Branch, Southeast University, Nanjing, China; ^2^ Department of Neonatology, Nanjing Lishui People’s Hospital, Zhongda Hospital Lishui Branch, Southeast University, Nanjing, China; ^3^ School of Medical Sciences, University of Sydney, Sydney, New South Wales, Australia; ^4^ School of Life Sciences, Huaiyin Normal University, Huai’an, China; ^5^ Department of Dermatology, Nanjing Lishui People’s Hospital, Zhongda Hospital Lishui Branch, Southeast University, Nanjing, China

**Keywords:** lung disease, lung ultrasonography, neonate, diagnosis, point of care

## Abstract

Lung disease is often life-threatening for both preterm and term newborns. Therefore, an accurate and rapid diagnosis of lung diseases in newborns is crucial, as management strategies differ with different etiologies. To reduce the risk of radiation exposure derived from the conventionally used chest x-ray as well as computed tomography scans, lung ultrasonography (LUS) has been introduced in clinical practice to identify and differentiate neonatal lung diseases because of its radiation-free characteristic, convenience, high accuracy, and low cost. In recent years, it has been proved that LUS exhibits high sensitivity and specificity for identifying various neonatal lung diseases. Here, we offer an updated review of the applications of LUS in neonatal lung diseases based on the reports published in recent years (2017 to present).

## Introduction

Caused by various factors, lung disease is a common condition that induces neonatal respiratory distress (1). Neonatal respiratory failure, which results from severe respiratory distress, accounts for a high proportion of neonatal deaths all over the world (2, 3). Therefore, clinicians, especially neonatologists, need to pay more attention to the identification and treatment of neonatal lung disease. Chest x-ray (CXR) and computed tomography (CT) scans are still conventionally used for diagnosing neonatal lung diseases. However, serious concern has been raised about the safety of various radiologic imaging examinations performed in diagnostic procedures, as they may lead to an increased incidence of malignancies (4, 5). In the neonatal intensive care unit (NICU), the most premature neonates are found to receive the highest radiation dose from diagnostic imaging procedures (6). The lack of a standard neonatal x-ray imaging practice at a global level poses a public health risk to neonates (7). Neonates and infants are highly susceptible to radiation toxicity. Therefore, radiation exposure during the neonatal period should be monitored, and alternative approaches without the risk of radiation exposure are needed in the NICU (8, 9). In addition, radiologic examination has various technical limitations and sometimes yields low accuracy (10).

As a powerful diagnostic and monitoring technique without ionizing radiation, ultrasonography has long been applied either for a superficial examination or for a study of deeply seated organs or systems. Traditionally, ultrasonography was not used to examine the lung because of the presence of lung air spaces in which ultrasonic waves are highly dissipated. However, this practice has now been broken, as lung ultrasonography (LUS) has been increasingly introduced in clinical practice since its first application in adult medicine in 1995 (11). As LUS is an accurate, reliable, quick, easy-to-use, real-time, low-cost, and radiation-free imaging modality, reports on its application in neonatology have notably increased during the last decade (12, 13). A 3-year clinical practice report published in 2020 indicated that CXR could be completely replaced by LUS in the NICU (14). Here, we aim to offer an updated review of recent advances and applications of LUS in neonatology and provide a better understanding of neonatal LUS.

## Examination technology and method

**Figure 1:**
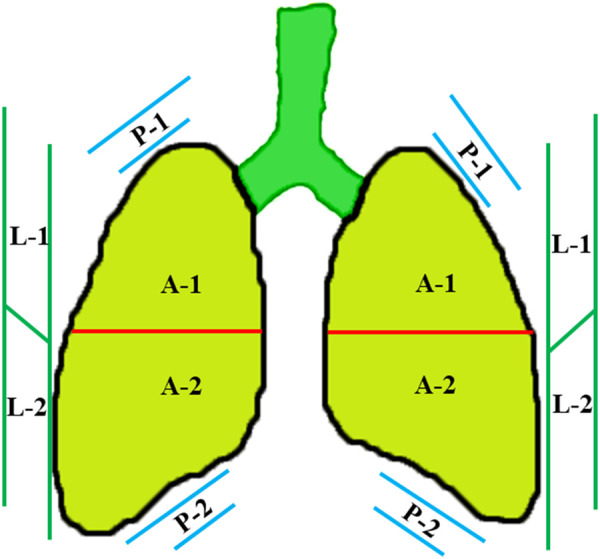
Divisions of the lung field. Each hemithorax can be separated into three regions by the anterior and posterior axillary lines, including the anterior (A) region, lateral (L) region, and posterior (P) region. In respect to each region, it can be further separated into an upper and a lower region.

In order to visualize the neonatal lung more effectively, a high-frequency (≥10 MHz) linear array transducer is suggested. For newborns bearing low gestational age (GA) and/or small birth weight, a higher-frequency probe can be applied. Under a quiet state, LUS examination can be performed in supine, prone, and side position for neonates. Based on the above positions, each hemithorax can be separated into three regions (Figure 1), namely the anterior (A) region, lateral (L) region, and posterior (L) region, by using the anterior and posterior axillary line. With respect to each area, it can be further separated into an upper and a lower region (15). For each region, longitudinal and transverse scans are applied by keeping the probe vertical to the ribs. In respect to the differential diagnosis of neonatal respiratory distress during the first 24 h of life, LUS and CXR exhibit a high concordance (16). Evidence-based guidelines for the use of point-of-care ultrasound (POCUS) in newborns and children provided by the European Society of Paediatric and Neonatal Intensive Care (ESPNIC) have an important role in standardizing the clinical practice of POCUS (17). An updated and detailed specification and guideline for neonatal LUS examination published this year has helped in the standardization of this examination. Therefore, it may be valuable in improving interobserver consistency in diagnostic accuracy, reliability, and patient outcomes (18).

## Normal neonatal LUS appearance

**Figure 2:**
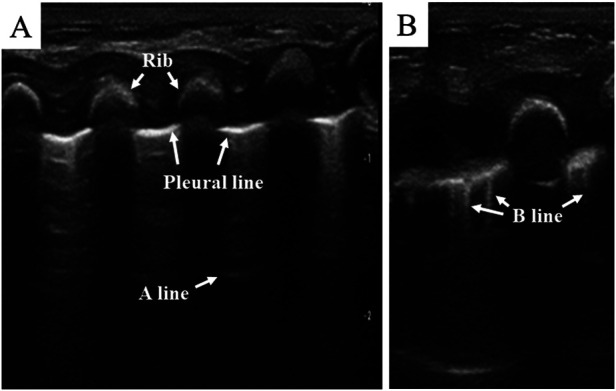
Normal lung pattern on lung ultrasonography. (**A**) Pleural line: the pleura is visualized as a smooth, regular echogenic line; A-lines: equidistant, horizontal, and echogenic lines distal to the pleural line. (**B**) B-line: vertical hyperechoic artifacts arising from the pleural line.

Normally, the ultrasound beam is dissipated by the lung air, which bears high acoustic impedance, and therefore, no acoustic mismatch can be obtained (19). However, as a result of the ultrasound reflected at the pleural–lung interface, the pleura is visualized as a smooth, regular echogenic line, called “pleural line” (Figure 2). In normal conditions, the pleural line thickness in healthy preterm and term newborns should be less than 1 mm (20), and the pleural line shifts to and fro synchronously with breathing. This movement, called “lung sliding” (11), is an indication of a healthy lung and can be easily detected by using a real-time ultrasound system. The repetitive reflection of the ultrasound waves between the pleura and the probe results in the formation of horizontal reverberation artifacts, called “A-lines” (Figure 2A). Parallel to the pleural line, the distal A-lines exhibit an equal interval from one another (19, 21). In addition, B-lines may be detected in normal lungs of neonates within 48 h after birth or longer in premature infants. These B-lines will disappear once the lung fluid is cleared (19, 21, 22). Derived from the ultrasound reflected by the alveolar air–liquid surface, B-lines vertically arise from the pleural line and extend to the screen's edge (Figure 2B). Unlike neonatal lungs, B-lines do not exist in normal children or adult lungs. Therefore, the obtained images containing A-lines as well as lung sliding indicate that there is no lung pathology in the scanned area.

## Neonatal LUS in diagnosis and description of specific lung diseases

### Respiratory distress syndrome

As a common cause of neonatal respiratory failure and early death, neonatal respiratory distress syndrome (RDS) is a manifestation of physiological and structural pulmonary immaturity. Therefore, it mainly occurs in those born prematurely (23). Pulmonary surfactant deficiency of prematurity has been identified as the primary cause of neonatal RDS (24). Pulmonary surfactant administration and mechanical ventilation are usually required to manage severe neonatal RDS (25).

**Figure 3:**
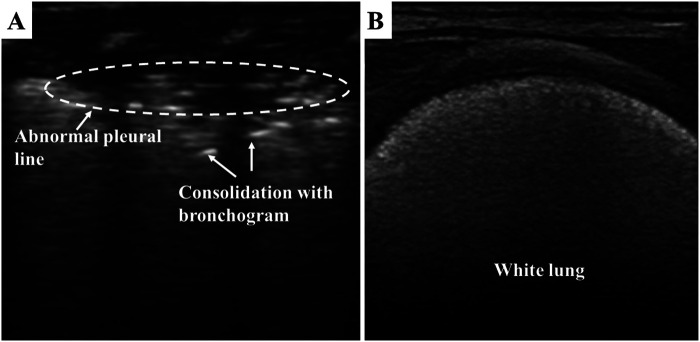
Respiratory distress syndrome on lung ultrasonography. (**A**) Pleural line is thick, irregular, or blurred. Lung consolidation with the air bronchogram is the most typical appearance of neonatal RDS. (**B**) white lung is derived from the emerged compact B-lines.

As shown in Figure 3, lung consolidation (hepatization of the lung) with air bronchogram is the most typical LUS appearance of neonatal RDS. The extent of lung consolidation of RDS usually depends on its severity (26). Longitudinal scans show a bilateral white lung (also known as alveolar-interstitial syndrome, AIS), which derives from the emerged compact B-lines observed in all the examined regions. The pleural line is thick, irregular, or blurred. In addition, pleural effusion and lung pulse may be detected in neonates with severe RDS (27, 28). Lung pulse occurs when the normal lung sliding disappears, and the pulmonary tissues beat synchronously with heart pulsation. It is an important sonographic sign of severe lung consolidation, such as atelectasis (29). It should be noted that the LUS appearance of neonatal RDS may be diverse between the bilateral lung and even in the different regions of the same side.

Based on a review of several related studies, LUS produces high sensitivity, specificity, and positive and negative predictive values in neonatal RDS diagnosis, and these values are consistently well above 90% (30–32). It has been confirmed that LUS is a reliable way to evaluate the severity of neonatal RDS (27) and to predict NICU admission in neonates with RDS (33). In addition, LUS has been applied in the follow-up of RDS, and therefore, unnecessary ionizing radiation can be reduced (34–36). As described above, surfactant administration is often needed for preterm babies with RDS. However, timely identification of neonates affected by RDS in need of surfactant therapy remains a challenge. In the current era, several LUS scores have been introduced as an effective predictor of the need for surfactant treatment among neonates with RDS (37–41). Based on the evaluation of treatment effect by LUS scores, poractant alfa produces a more favorable outcome than beractant (42), and the treatment effect of poractant alfa and beractant is superior compared with that of calfactant (43). These positive results indicate that LUS shows promise as a useful tool for decision-making during the treatment of neonatal RDS (44, 45). However, these studies still have various limitations. Thus, large multicenter and well-designed studies are needed prior to using the LUS score as a regular tool for the prediction of surfactant treatment in newborns with RDS (46, 47).

### Transient tachypnea of the newborn

As another causative factor for dyspnea in neonates, transient tachypnea of the newborn (TTN) or “wet lung” results from delayed lung fluid clearance. In normal conditions, LUS video recordings show that substantial liquid clearance occurs within the first 20 min, and complete clearance is accomplished within the first 4 h after birth (19, 21, 48). Compared with vaginal delivery, an elective cesarean section may lead to a delayed fluid clearance within the first 3 h of birth (49). In addition, immature GA, influencing the functions of ENac, is also one of the causes of TTN (50). In most cases, TTN presents as a mild or transient respiratory distress, which will improve within 24–48 h. However, symptoms may persist for a long time in some severe cases.

**Figure 4:**
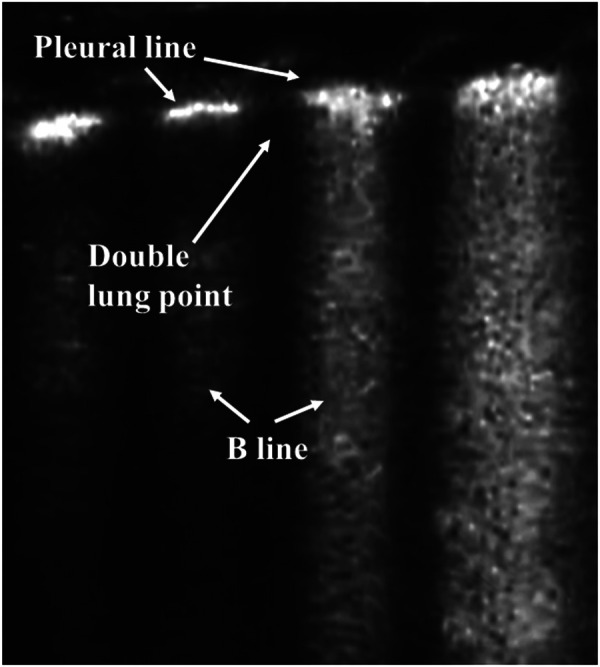
Transient tachypnea of the newborn on lung ultrasonography. Double lung point: there is a sharp change in echogenicity as a result of the abundance of B-lines between the upper and the lower lung fields.

As a result of fluid retention in the lung tissues, pulmonary edema is the common characteristic of TTN. By evaluating the distribution of B-lines, the primary LUS characteristics of TTN, which are related to its severity, include the double lung point (DLP), AIS, or white lung. The DLP means that there is a sharp change in echogenicity as a result of the abundance of B-lines between the upper and the lower lung fields (Figure 4) (48, 51). These appearances are evident bilaterally, but not always symmetrically. In most cases of TTN, the pleural line is regular, and no significant subpleural lung consolidation is present (52). This consistent finding in TTN may be used to rule out other lung disorders such as RDS and pneumonia. The DLP alone is not sufficient reason for performing a diagnosis of TTN. However, LUS can achieve good diagnostic accuracy in detecting TTN when a combination of the DLP and the B-line is applied (53–56). Therefore, exclusive diagnosis is the common method to identify TTN. However, compared with traditional CXR, which exhibits a low sensitivity and specificity for detecting pulmonary edema, LUS performs better in TTN diagnosis (26, 57, 58). Furthermore, LUS can effectively differentiate TTN from RDS (32, 59–61), which is important for providing correct treatment for neonates with respiratory distress. Therefore, LUS is accurate and reliable in diagnosing TTN.

### Pneumonia

As a pulmonary inflammatory process, pneumonia leads to significant morbidity and mortality during the neonatal phase, especially in developing countries (62, 63). Epidemiological data indicate that the prevalence of neonatal pneumonia is more severe than presumed (64). Approximately 14% of newborns had experienced pneumonia in China, and potential risk factors contributing to the development of neonatal pneumonia were identified, such as limited or no antenatal care, home delivery, fever at birth, gynecological diseases during pregnancy, and prolonged labor (65). Various bacteria, viruses, or fungi are common pathogens that can induce neonatal pneumonia by intrauterine or postnatal routes (63). Respiratory distress is the common sign of neonatal pneumonia. However, its radiographic findings are often non-specific, which leads to the fact that it may be indistinguishable from RDS and TTN. Therefore, to better determine the type of pneumonia, clinical laboratory findings (such as routine blood tests, C-reactive protein, and blood culture) are needed.

**Figure 5:**
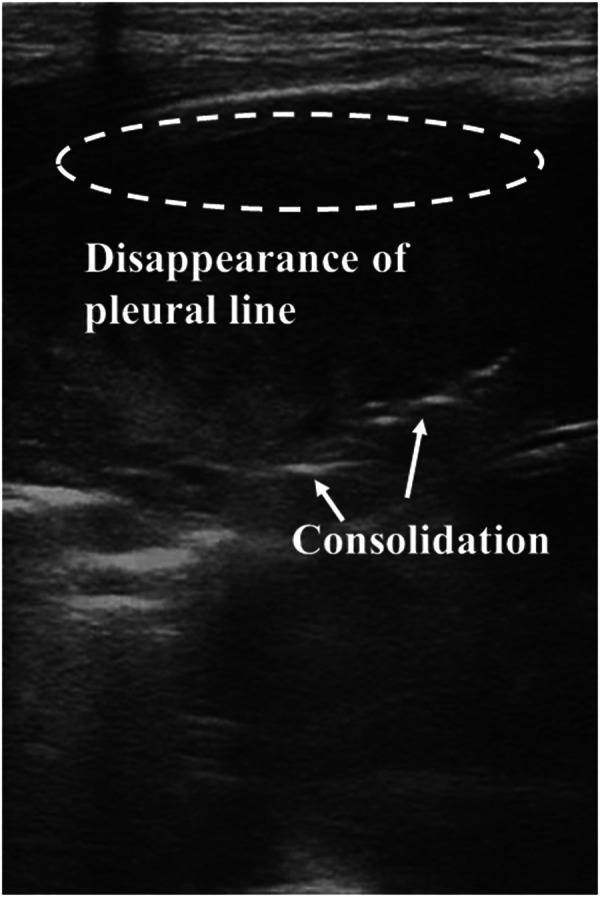
Pneumonia on lung ultrasonography. Ultrasound shows irregular areas of lung consolidation with air bronchograms, disappearance of the pleural line, and A-lines.

LUS shows promise in diagnosing pneumonia in adults (66) and children (67). These typical LUS appearances of pneumonia mainly include subpleural consolidations with irregular margins and air bronchograms, irregular pleural lines, absent lung sliding, and pleural effusion (68–70). However, reports analyzing the effect of LUS on diagnosing neonatal pneumonia are limited. In a study reported in 2013, LUS exhibited high sensitivity, specificity, and reproducibility in diagnosing neonatal pneumonia (71). A sensitivity of 100% and a specificity of 100% were obtained by applying the presence of large consolidated regions with irregular borders and air bronchograms (Figure 5) as one of the diagnostic criteria for neonatal pneumonia (72). Subsequently, the same research group obtained similar results by introducing LUS into the NICU (26). Based on the detection of ultrasonographic changes in patients before treatment and during discharge (such as the presence or absence of consolidation with air bronchograms, pleural line abnormalities, and pleural effusion), LUS is seen as an effective technique in the diagnosis and monitoring of neonatal viral pneumonia (73). For newborns with infectious pneumonia, the LUS score can be applied to evaluate the severity of the disease, and they are negatively correlated (74). Based on the proposed multiparameter ventilator-associated pneumonia (VAP) score, LUS can be considered an alternative to CXR to diagnose neonatal VAP (75).

Coronavirus Disease 2019 (COVID-19), caused by Severe Acute Respiratory Syndrome Coronavirus-2 (SARS-Cov-2), first occurred in China at the end of 2019 and has since spread to many countries and posed a serious threat to the entire world (76). Apart from being infected by people who have tested positive for SARS-Cov-2, newborn children may catch infection from a vertical transmission in mothers with COVID-19, which, however, has yet to be confirmed (77, 78). LUS has been applied as a method for diagnosing neonatal COVID-19. The related LUS appearances include irregular pleural lines, increased and compact B-lines, and AIS and may coexist with small areas of lung consolidation (77, 79–81). Compared with CXR and CT, LUS exhibits a better sensitivity and safety in diagnosing neonatal COVID-19. Therefore, LUS may be used as an effective method to monitor and evaluate lung lesions in neonates with COVID-19 (82, 83). This acquires more importance in under-resourced areas. Wireless ultrasound probes can be used to reduce the risk of contamination, thus protecting the patient and the physician (84). Thus, LUS is a reliable tool to diagnose and evaluate neonatal pneumonia.

### Meconium aspiration syndrome

Caused by antepartum or puerperal inhalation of meconium-stained amniotic fluid, meconium aspiration syndrome (MAS) mainly occurs in term/post-term neonates, leading to respiratory distress of varying severity. The etiology of MAS is complex, and various risk factors such as birth asphyxia and cesarean delivery have been identified for the development of MAS (85, 86). Clinical manifestations, laboratory tests, and radiographic findings can be used to diagnose MAS. Symptomatic and supportive approaches such as mechanical ventilation and surfactant administration are commonly needed to treat MAS (87).

Based on limited reports, LUS appearances of MAS show similarity to that of pneumonia, including irregular subpleural consolidations with air bronchogram, coalescent or sparse B-lines, an abnormal pleural line, and the disappearance of the A-line (88, 89). LUS was performed on six neonates with MAS of variable severity during their first hours of life. Coalescent or sparse B-lines, consolidation, atelectasis, and bronchograms were found, and these results corresponded well with CXR findings (90). In a relatively large-scale study comprising 117 neonates with MAS and 100 controls, similar LUS characteristics were detected, and irregular subpleural consolidations with air bronchograms showed 100% sensitivity and specificity in diagnosing MAS (91). These ultrasonographic findings are not specific to MAS. Particularly, irregular pulmonary consolidations can also be found in cases of pneumonia as described above. However, consolidations are usually found bilaterally in MAS but only on one side in pneumonia. Therefore, it is relatively easy to differentiate between MAS and pneumonia (89).

### Air leak syndromes

When air escapes from the alveoli to the extra-alveolar regions, air leak syndromes are formed. Various air leak syndromes (for example, pneumothorax, pneumomediastinum, and pulmonary interstitial emphysema) can be found in neonates. The most common reason for inducing these related syndromes in neonates is inappropriate mechanical ventilation toward the fragile and immature lungs. Thus, these syndromes occur more frequently during the neonatal phase than at any other age (92). Among these air leak syndromes that occur in neonates, pneumothorax is the most common one, and 1%–2% of all term neonates may exhibit spontaneous pneumothorax. However, this incidence may go up to 30% in individuals with various lung pathologies or requiring mechanical ventilation (93). Tube thoracostomy is a safe and effective procedure in the management of pneumothorax (94). Traditionally, CXR or CT has played an important role in diagnosing pneumothorax. In recent years, LUS has been successfully applied in diagnosing neonatal pneumothorax.

**Figure 6:**
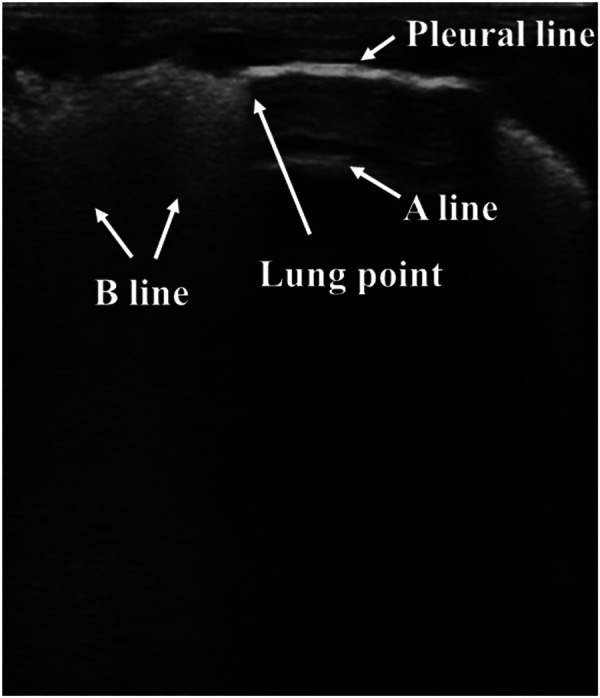
Pneumothorax on lung ultrasonography. Lung point: the transition from the B-lines area (left side) to a hypoechoic area (right side).

On ultrasound, pneumothorax exhibits no lung sliding, no B-lines, clear A-lines, and the presence of the lung point. The air present in the pleural cavity abolishes lung sliding and B-lines. The lung point refers to the interface between a normal pattern with lung sliding and an abnormal pattern without lung sliding (Figure 6). Therefore, the lung point represents the physical border of pneumothorax. As there is too much air in the chest cavity, the highly compressed lung deviates from the chest wall. Therefore, the lung point may not be present in these severe cases. However, the lung point is still a critical and specific characteristic of mild to moderate pneumothorax (95, 96). In adults, a meta-analysis has shown that LUS exhibits a better diagnostic accuracy for pneumothorax than traditional chest radiography (97). A similar advantage of LUS in diagnosing pneumothorax in neonates has also been found in relatively limited studies. A sensitivity of 100% and a similar rate of specificity of LUS for the diagnosis of pneumothorax in neonates were detected. LUS is at least as reliable as CXR in diagnosing neonatal pneumothorax (98–100). In a recent study, it was found that the sensitivities of absent B-lines, absent lung sliding, as well as the presence of the lung point in diagnosing pneumothorax in newborns were 100%, 100%, and 94.28%, respectively, and all the related specificities reached 100%. Compared with CXR, LUS exhibited better sensitivity and specificity in early detection of neonatal pneumothorax (101). Compared with the “mirrored ribs” sign, which lacks specificity for neonatal pneumothorax as can often be observed in other situations or in lungs without pneumothorax, A-lines in the anterior transverse plane show high diagnostic utility in diagnosing pneumothorax (102). Combined with other case reports of using LUS in diagnosing pneumothorax (103, 104) and the proposed procedures for diagnosing pneumothorax by LUS (89), LUS is considered accurate and reliable for diagnosing neonatal pneumothorax. Therefore, these limited data support the opinion that LUS is better than or at least as accurate as CXR in diagnosing neonatal pneumothorax, thus highlighting its treatment efficacy. However, prospective studies are needed to further investigate the practical clinical value of LUS in diagnosing neonatal pneumothorax.

For neonatal localized interstitial emphysema, its LUS appearances show bilateral coalescent B-lines and pleural thickness without spared regions. It has been proposed that LUS shows promise as a useful tool for its identification and follow-up (105). LUS examination performed on one neonate with pneumomediastinum (day 2 of life) exhibits multiple A-lines, absence of B-lines, and no pleural shimmering. At the same time, the classic “bar-code” sign can be observed using M-mode ultrasound (106). Thus, it is believed that LUS shows potential to be used as a promising imaging approach to detect pneumomediastinum in newborns.

### Bronchopulmonary dysplasia

As a major complication of prematurity, bronchopulmonary dysplasia (BPD) is a common chronic respiratory morbidity seen in one-third of extremely low-birth-weight babies (107). However, it is still challenging to give a standardized definition for BPD, and various definitions have been applied in the literature, leading to broad variations in reporting the incidence of BPD. Among neonates born <28 weeks gestation, this incidence is estimated to be 48%–68%, which significantly enhances along with declining GA (108). The imbalance between lung injury, which can be caused by various factors, and repair in the developing immature lung leads to the development of BPD. Various risk factors occurring during the antenatal, natal, or postnatal period may contribute to BPD (109). As there is no known cure for BPD, infants with BPD often need long-term oxygen treatment, leading to possible lifelong variations in cardiopulmonary activity (110). Various potential cytokines and proteins (111, 112) and chest radiographs (113) have been studied as possible predictors for BPD. However, none of them has been routinely applied in the clinic. Early prediction of BPD is also a potentially interesting application of LUS.

**Figure 7:**
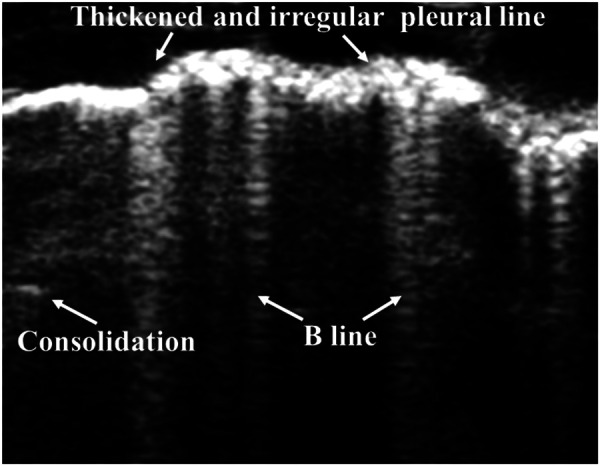
Bronchopulmonary dysplasia on lung ultrasonography. Ultrasound shows unequally distributed B-lines, thickened pleural lines, and multiple different-sized subpleural consolidations.

For LUS images of BPD, patterns of unequally distributed B-lines, accompanied by thickened pleural lines and multiple different-sized subpleural consolidations, can be observed (Figure 7) (88, 114, 115). Hyperoxia-exposed preterm rabbits exhibit an LUS appearance similar to human preterm neonates with evolving BPD, and this is valuable in LUS training (116). Persistent retrodiaphragmatic hyperechogenicity detected by LUS with a transabdominal probe has been shown to be capable of predicting the development of BPD (117, 118). Among 50 premature babies clinically identified with BPD, LUS examinations show that more than one-third of them exhibited various other lung pathologies (119). This suggests that a revision of the current diagnostic criteria for BPD, which depend primarily on the length of time of oxygen dependence and may result in an overdiagnosis, is needed. A semiquantitative LUS score has been shown as an effective predictor of BPD. At 1–2 weeks of age, it can predict the diagnosis of BPD, and moderate to severe BPD may be predicted at 4 weeks of age (120). Subsequently, various trials aimed at evaluating the predictive ability of LUS in neonatal BPD have been reported. In preterm neonates with RDS, the LUS pattern is gestational age-dependent and helps to predict BPD in the 28–30-week postmenstrual age (PMA) (121). Among these neonates of <29 weeks of gestation, LUS scores obtained during the first 2 weeks (at days 3, 7, and 14) after birth reveal an excellent prediction of BPD, and the LUS alone is found to be better in predicting BPD than the combined clinical variables related to the prediction of BPD (122). Several reports have shown that LUS scores can predict the occurrence of BPD in preterm neonates, starting from the 7th day of life (DOL) (123–125). The diagnostic accuracy of predicting BPD on the 3rd and 7th DOL can be improved by adding the factors of sex and GA (126). Similarly, when combined with clinical variables, the prediction time of BPD can be brought forward to the 6th DOL compared with the LUS alone (9th to 15th DOLs) (127). A recent meta-analysis on this issue confirms that LUS scores are accurate for the early prediction of BPD in preterm neonates of ≤32 weeks of gestation (128). Based on its convenience and radiation-free feature, LUS is valuable in evaluating the severity of neonatal BPD, therefore providing clues to precision treatment for BPD neonates (115). In summary, LUS, especially when combined with clinical variables related to BPD, is a highly predictive and reproducible diagnostic tool for early prediction of BPD in preterm neonates. Therefore, the application of LUS scores in predicting BPD should be advocated. In addition, larger multicentric prospective studies and more validation studies are needed to determine the optimal LUS scoring procedure.

Thus, LUS shows promise as a predictor of BPD. However, further related research is needed to buttress this finding.

### Atelectasis

As a state of collapse and failed aeration of the lung parenchyma, atelectasis presents as a common occurrence in neonates. It can lead to neonatal dyspnea and prolonged illness (129). Atelectasis is a common complication of various pulmonary and chest disorders, including RDS, MAS, and pneumonia, rather than an independent disease. Three different mechanisms accounting for atelectasis have been proposed (129). Airway obstruction is the most common risk factor for atelectasis in infants. Long-term atelectasis may induce other complications such as secondary infection and bronchial damage, and various treatments can be applied according to its cause, duration, and severity (89, 129). The commonly used diagnostic methods for atelectasis include CXR, CT, and bronchoscopy. However, LUS has also been used for diagnosing neonatal lung atelectasis.

**Figure 8:**
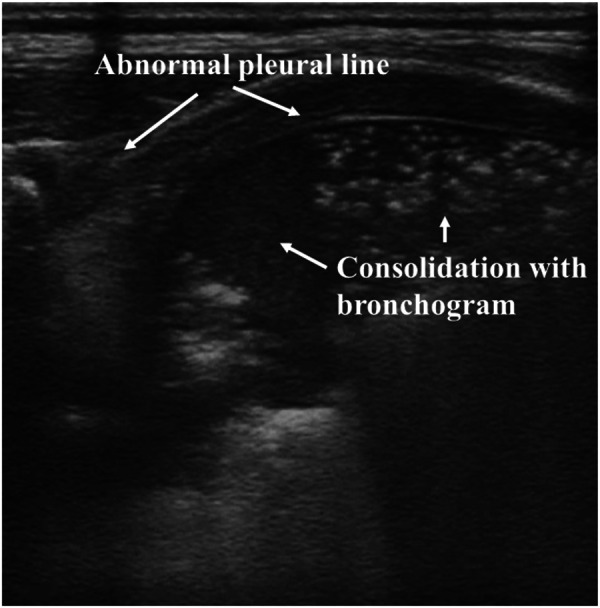
Atelectasis on lung ultrasonography. Ultrasound shows abnormal pleural lines, absent A-lines, and large areas of consolidation.

On ultrasound, atelectasis exhibits extensive lung consolidation with bronchograms, an abnormal pleural line, no A-lines, no lung sliding, as well as the presence of lung pulse (Figure 8). Compared with the consolidation present in pneumonia, the large areas of consolidation present in atelectasis act as well-defined borders (Figure 8). Combined with CXR and CT, LUS can confirm the presence of neonatal atelectasis, and subsequently, LUS plays a major role in monitoring disease development, without exposing patients to ionizing radiation (130). In a study that enrolled 80 newborns with atelectasis and 50 normal controls, LUS showed 100% sensitivity in diagnosing atelectasis. However, the related sensitivity of CXR was only 75%. Also, large consolidations with clear borders exhibited 100% specificity for neonatal atelectasis (131). In addition, bronchoalveolar lavage under ultrasound monitoring showed high effectiveness in treating neonatal atelectasis without adverse side effects (132). Therefore, LUS is capable of diagnosing neonatal atelectasis accurately and reliably.

## LUS in other neonatal lung diseases

Apart from these specific neonatal lung diseases described above, LUS has also been reported to diagnose other neonatal lung disorders, which are given in the following paragraphs.

### Pulmonary hemorrhage

As a common severe disease in neonates, pulmonary hemorrhage has a high mortality rate, and its rapid and accurate diagnosis is essential for treatment (133). Two reports from the same research group have indicated that LUS exhibits the ability to diagnose neonatal pulmonary hemorrhage (134, 135). Based on the obtained LUS findings, the shred sign, which locates the edge of the consolidation area, possesses a high sensitivity and specificity for diagnosing pulmonary hemorrhage.

### Congenital diaphragmatic hernia

Congenital diaphragmatic hernia (CDH), which occurs approximately 2.5 in 10,000 births, induces various levels of pulmonary hypoplasia and pulmonary hypertension (136). It has been reported by two case reports (137, 138) that point-of-care LUS can exclude cardiac disease and pneumothorax rapidly and expedite the diagnosis of CDH. Also, this positive role of LUS in diagnosing CDH has been confirmed by describing LUS patterns in infants with this pathology (139).

### Congenital pulmonary airway malformation

Comprising different congenital disorders of the lung and airways, congenital pulmonary airway malformation (CPAM) exhibits an incidence of approximately 1 in 10,000 to 1 in 35,000 births. It causes significant mortality and morbidity in infants (140). The introduction of LUS appearances in newborns with CPAM was first reported in 2018. LUS shows potential as an imaging approach to confirm prenatally identified CPAM, and it can be suspected once cystic lung lesions are observed by LUS in infants with respiratory distress (141). The effectiveness (accuracy and safety) of LUS in the management of neonatal CPAM has been proved by subsequent reports (142, 143). As a result of the high level of consistency with CT, LUS may be used routinely to confirm the diagnosis of neonatal CPAM and for follow-up and management. In addition, a recent report based on a single-center, retrospective cohort study confirms that LUS is valuable in the preliminary qualitative screening of CPAM in infants, and the diagnostic performance of the indirect signs of LUS is better than that of CXR (144).

### Bronchiolitis

As a lower respiratory tract disease, bronchiolitis usually occurs in the first life year. A systemic review suggests that LUS aids the clinical management of bronchiolitis in young children by, for example, reducing unnecessary exposure to ionizing radiation and avoiding the needless administration of antibiotics (145). However, its role in neonatal bronchiolitis needs further study.

### Congenital chylothorax

Although it is a rare entity, congenital chylothorax is the most common reason for pleural effusion in neonates, and various treatment strategies have been introduced and implemented (146, 147). It has been suggested that LUS should be considered for the diagnosis, monitoring, and follow-up of neonates with congenital chylothorax (147, 148).

## Other applications beyond diagnosis

Apart from being used as a diagnostic method for neonatal lung diseases, as described above, LUS can play a more “functional” role in guiding or assisting treatments for neonatal lung diseases through real-time monitoring and assessment. One of the most important applications is the recently proposed use of the LUS score as a semiquantitative assessment of the severity of lung diseases. Derived from LUS scores applied in adults, which have been used to assess pulmonary aeration as well as oxygenation, a modified LUS score for neonates was proposed, and it possesses a good correlation with oxygenation status in newborns. Therefore, it can be used to predict the need for surfactants in preterm infants (149). Its positive role in predicting surfactant need in very- and extremely preterm newborns (39, 150) and also the development of chronic lung disease in preterm neonates (151) has been reported recently. However, the role (for example, accuracy) of the semiquantitative LUS scores in evaluating the severity of neonatal lung diseases is still a matter of debate because, currently, two senior medical experts hold the opposite opinion (152, 153).

LUS scores obtained during the first days of life can provide information about the prognosis of neonatal respiratory failure (154) and predict the need for respiratory support (155–157). In addition, LUS performs a useful role in predicting non-invasive ventilation failure in neonates with respiratory distress, and this is important for the clinician to decide to apply invasive mechanical ventilation to prevent clinical deterioration (154, 158–160). Based on the obtained LUS severity score, point-of-care LUS can predict the weaning readiness off nasal continuous positive airway pressure (NCPAP) in premature infants with evolving BPD (161) and also the success of extubation among ventilated neonates (162–164), thus avoiding their exposure to multiple unsuccessful weaning cycles. As described above, surfactant replacement therapy may be needed in neonates with serious respiratory disease, and early identification of this need is important. The only randomized clinical trial on this issue reported so far indicates that the LUS score improves timeliness in surfactant replacement compared with using FiO_2_ criteria alone (165). The accuracy of LUS for predicting the need for surfactant replacement increases when combined with the oxygenation index, and this is valuable in clinical decision-making (166, 167). A critical systemic review and meta-analysis has shown that the LUS score could be employed accurately to predict the need for surfactant treatment in early preterm infants with respiratory distress treated with NCPAP (168). In addition, an investigation indicates that LUS-guided surfactant administration in preterm neonates with RDS does not increase pharmaceutical expenditure, including the cost of surfactant therapy and global surfactant utilization (169).

It has been reported that LUS can be used to guide pulmonary recruitment maneuvers during mechanical ventilation in neonates (170, 171). As the developed artifacts resemble sunrays crossing the clouds, the novel S-lines (“sunray lines”) described in the LUS-guided protocol may improve the efficacy and safety of mechanical ventilation strategies in neonates (170). In addition, computer-aided image analyses have been developed to interpret LUS findings. In recent years, various versions of quantitative ultrasound fetal lung maturity analysis software (quantusFLM) have been reported to be used as an automated and non-invasive approach for the prediction of neonatal respiratory morbidity with a performance result similar to that of tests using amniotic fluid (172–174). Point-of-care LUS has also played an important role in guiding or assisting neonatal pulmonary disease management such as guiding bronchoalveolar lavage to treat neonatal atelectasis, MAS, and severe pneumonia, guiding thoracentesis to treat massive pleural effusion and pneumothorax, guiding the application of neonatal mechanical ventilation, and evaluating the effects of surfactant administration (175).

## Conclusion

Owing to its radiation-free, convenient, non-invasive, accurate, and less expensive characteristics, LUS has played an important role in the identification and differential diagnosis of various neonatal pulmonary disorders. In addition, because of its easy operation and its commercially automated and non-invasive software, it is worthy and necessary to extend the clinical applications of LUS in neonatal lung diseases further.
